# Urge intolerance predicts tic severity and impairment among adults with Tourette syndrome and chronic tic disorders

**DOI:** 10.3389/fpsyt.2022.929413

**Published:** 2022-08-10

**Authors:** Kesley A. Ramsey, Alessandro S. De Nadai, Flint M. Espil, Emily Ricketts, Jordan T. Stiede, Jennifer Schild, Matthew W. Specht, Douglas W. Woods, Shannon Bennet, John T. Walkup, Susanna Chang, John Piacentini, Joseph F. McGuire

**Affiliations:** ^1^Division of Child and Adolescent Psychiatry, Department of Psychiatry and Behavioral Sciences, Center for OCD, Anxiety, and Related Disorders for Children (COACH), Johns Hopkins University School of Medicine, Baltimore, MD, United States; ^2^Department of Psychology, Texas State University, San Marcos, TX, United States; ^3^Department of Psychiatry and Behavioral Sciences, Stanford University School of Medicine, Stanford, CA, United States; ^4^Division of Child and Adolescent Psychiatry, UCLA Semel Institute for Neuroscience and Human Behavior, Los Angeles, CA, United States; ^5^Department of Psychology, Behavior Therapy and Research Lab, Marquette University, Milwaukee, WI, United States; ^6^Department of Psychology, Choices Youth Psychopathology Lab, Suffolk University, Boston, MA, United States; ^7^Department of Psychiatry, Weill-Cornell Medicine, New York, NY, United States; ^8^Department of Psychiatry and Behavioral Sciences, Northwestern University Feinberg School of Medicine, Chicago, IL, United States

**Keywords:** Tourette Syndrome, premonitory urge, distress tolerance, adults, impairment

## Abstract

**Background:**

Individuals with Tourette Syndrome and Persistent Tic Disorders (collectively TS) often experience premonitory urges—aversive physical sensations that precede tics and are temporarily relieved by tic expression. The relationship between tics and premonitory urges plays a key role in the neurobehavioral treatment model of TS, which underlies first-line treatments such as the Comprehensive Behavioral Intervention for Tics (CBIT). Despite the efficacy of CBIT and related behavioral therapies, less than 40% of adults with TS respond to these treatments. Further examination of the relationship between premonitory urges, tic severity, and tic impairment can provide new insights into therapeutic targets to optimize behavioral treatment outcomes. This study examined whether urge intolerance—difficulty tolerating premonitory urges—predicted tic severity and tic-related impairment among adults with TS.

**Methods:**

Participants were 80 adults with TS. Assessments characterized premonitory urge, distress tolerance, tic severity, and tic impairment. We used structural equation modeling (SEM) to examine the construct of urge intolerance—comprised of premonitory urge ratings and distress tolerance ratings. We first evaluated a measurement model of urge intolerance through bifactor modeling, including tests of the incremental value of subfactors that reflect premonitory urge severity and distress tolerance within the model. We then evaluated a structural model where we predicted clinician-rated tic severity and tic impairment by the latent variable of urge intolerance established in our measurement model.

**Results:**

Analyses supported a bifactor measurement model of urge intolerance among adults with TS. Consistent with theoretical models, higher levels of urge intolerance predicted greater levels of clinician-rated tic severity and tic impairment.

**Conclusion:**

This investigation supports the construct of urge intolerance among adults with TS and distinguishes it from subcomponents of urge severity and distress tolerance. Given its predictive relationship with tic severity and tic impairment, urge intolerance represents a promising treatment target to improve therapeutic outcomes in adults with TS.

## Introduction

Tourette Syndrome and other persistent tic disorders (collectively referred to as TS) are neuropsychiatric conditions characterized by the recurrence of sudden, involuntary motor and vocal tics. Prevalence estimates suggest that TS affects ≈ 1% of youth, and symptoms often persist into adulthood for many patients (1–3). In addition to tics, individuals with TS often experience a variety of comorbid psychiatric conditions [e.g., attention deficit hyperactivity disorder (ADHD), obsessive-compulsive disorder (OCD), anxiety disorders, depressive disorders] and co-occurring challenges with affect and behavioral regulation (e.g., suicidality, affect lability) (4–8). Tics, accompanying premonitory urges, and co-occurring psychiatric conditions contribute to significant impairment for individuals with TS across the lifespan (9–15). Behavioral therapies—such as habit reversal training (HRT), the Comprehensive Behavioral Intervention for Tics (CBIT), and Exposure with Response Prevention (ERP)—have emerged as first-line interventions for individuals with TS (16–18). For individuals who exhibit a positive response to behavioral treatments, therapeutic gains are maintained for over 6 months (19, 20) and can have lasting benefits for up to 11 years (21). Despite the benefit of behavioral treatments for some adults with TS, less than 40% respond to this treatment approach (22). Thus, there is a critical need to understand factors that influence treatment response to evidence-based behavioral therapies in this age group, which can ultimately lead to the identification of novel therapeutic targets that optimize treatment outcomes (23, 24).

Behavior therapy for TS is grounded within a neurobehavioral model of tics. While this model acknowledges neurobiological contributors (e.g., neurotransmitters, brain circuitry, genetics), it suggests that tic expression is influenced by external (e.g., environmental context) and internal factors (e.g., premonitory urge, affective states) (25, 26). These internal and external factors serve as primary targets of intervention in behavior therapy (25). For instance, premonitory urges serve as antecedents to tics and are alleviated by tic expression, which in turn create a negative reinforcement cycle thought to maintain tic expression (27). In behavior therapy, individuals with TS learn to build awareness to tics and associated antecedents (e.g., urges) and implement competing responses to inhibit tics contingent upon antecedents (25, 26). Consequently, greater distress tolerance of premonitory urges would likely allow individuals to effectively implement competing responses even during intense premonitory urges, and therefore be associated with better behavioral therapy outcomes (e.g., reductions in tic severity and tic impairment). To date, the inability to tolerate premonitory urges (i.e., urge intolerance) has received limited investigation (28). Although the precise mechanisms underlying behavioral therapies are not fully explicated (26), urge intolerance represents an important construct that warrants further investigation.

The construct of urge intolerance is comprised of two central features: premonitory urge severity and distress tolerance. At present, no rating scales have been designed to specifically measure individuals' intolerance of urge sensations. In the absence of specific rating scales, existing validated rating scales (i.e., Premonitory Urge for Tics Scale [PUTS], Distress Tolerance Scale [DTS]) can be combined to understand this clinically-relevant construct. Indeed, prior work has started to explore urge intolerance (a latent variable derived from combined PUTS and DTS ratings) among youth with TS, and found that greater levels of urge intolerance predicted greater levels of parent- and child-reported functional impairment (28). However, further research is essential to understand the construct of urge intolerance across the lifespan, which may potentially explain the different rates of treatment response to behavior therapy between youth and adults.

Accordingly, this study investigated urge intolerance in adults with TS. First, structural equation modeling was used to build and test models of urge intolerance using validated rating scales. We hypothesized that a bifactor model of the latent construct of urge intolerance, comprised of urge severity and distress tolerance, would demonstrate good model fit. Second, the relationship between the latent construct urge intolerance and clinician-rated tic severity and tic impairment on the Yale Global Tic Severity Scale (YGTSS) was examined. We anticipated that greater levels of urge intolerance would predict greater levels of clinician-rated tic severity and tic impairment among adults with TS.

## Method

### Participants

The present sample included 80 adults with TS who participated in a 11.17-year (SD = 1.25) long-term follow-up assessment for a randomized clinical trial of behavior therapy for tics in youth with TS (21, 29). Participants needed to be enrolled in the original clinical trial of behavior therapy to participate in this long-term follow-up assessment. There were no significant differences on demographic and clinical characteristics between participants who completed the long-term follow-up assessment, those who declined to participate in the long-term follow-up assessment, and those who were lost to follow-up [see Espil et al. (21) for further details].

Participants were 23 years of age on average (*M* = 22.87, *SD* = 2.70), predominantly male (*n* = 60, 75%), and mostly Caucasian (*n* = 69, 86%). Most participants met criteria for a diagnosis of Tourette's disorder (*n* = 74, 92%), while other participants met criteria for a current diagnosis of chronic motor tic disorder (*n* = 6, 8%). Common co-occurring conditions among participants included: anxiety disorders (*n* = 18, 23%), ADHD (*n* = 11, 14%), and OCD (*n* = 7, 9%). Less than one-third of participants (*n* = 8, 29%) were taking medication for tic management (e.g., antipsychotic or alpha-2 adrenergic agonist medication).

### Measures

Yale global tic severity scale (YGTSS) (30). The YGTSS is a clinician-administered assessment that measures tic severity in the past week across five domains: number, frequency, intensity, complexity, and interference domains (30). Item ratings are summed for motor and vocal tics to produce a Total Tic Severity score (range: 0–50). Clinicians also record a global rating for tic-related impairment in the past week (range: 0–50). The YGTSS has shown good reliability and validity across studies (30–32).

Premonitory urge for tics scale (PUTS) (33). The PUTS is a 9-item self-report questionnaire that measures premonitory urge phenomena (33). Items inquire about the frequency and discomfort associated with premonitory urges, and are rated on a 4-point scale. Items are summed to produce a total score (range: 0–36), with higher scores indicative of greater levels of premonitory urge severity. The PUTS has good internal consistency and external validity across individuals with TS (34, 35).

Distress tolerance scale (DTS) (36). The DTS is a 15-item self-report questionnaire that assesses an individual's ability to tolerate distress (36). Items are rated on a 5-point scale, and are summed to yield a total score (range: 15–75). Higher total score values indicate less distress tolerance. The DTS has demonstrated good convergent and divergent validity (36).

### Procedures

All procedures followed ethical standards for human subject research and were approved by local institutional review boards (IRBs). Participants from the original clinical trial were contacted to participate in a long-term follow-up assessment (21, 29). Eighty participants (i.e., 63.4% of the original sample) were interviewed in-person or via Skype by trained raters to ascertain clinical history and psychiatric diagnoses on the Mini-International Neuropsychiatric Interview (37). Next, clinician-administered assessments were completed to characterize current tic severity (YGTSS). Finally, participants completed self-report measures of premonitory urges (PUTS) and distress tolerance (DTS). Please see Espil et al. (21) for further details.

### Analytic plan

Descriptive statistics and correlations characterized the sample and associations between relevant clinical constructs. Structural equation modeling (SEM) in Mplus examined the construct of urge intolerance using items from the PUTS and DTS (38). SEM is ideal for investigating latent theoretical constructs that cannot yet be directly measured or observed (39). Additionally, SEM allows for the further exploration of relationships between a latent construct and other observed characteristics.

A bifactor structural model was selected to measure the latent construct of urge intolerance. A bifactor approach specifies that the covariance among a set of items can be accounted for by a single, general factor that captures the common variance among all items in the set, while also allowing for subfactors to explain item subgroups (40). A bifactor model approach is recommended when there is a strong justification for capturing a superordinate construct along with distinct subordinate constructs. The bifactor model confers several statistical advantages. In addition to better specifying the model (i.e., delineating general and specific subfactors within a single model), this approach allows for simultaneous evaluation of item loading on both the general factor (i.e., urge intolerance) and unique subfactors (PUTS, DTS) (41). In order to evaluate the fit of the hypothesized bifactor model of urge intolerance with its corresponding urge and distress tolerance subfactors, the incremental value of including distinct subfactors of premonitory urge severity and distress tolerance within the model was examined. To evaluate the incremental value of each component of the model, nested models were compared through adjusted likelihood ratio tests (42). In the first step, we evaluated model fit for a full bifactor model, comprised of the PUTS and DTS items loading onto the general urge intolerance factor, as well as their respective urge severity and distress tolerance subfactors. In the second step, a constrained version of the bifactor model was evaluated, with the general latent factor urge intolerance fixed at 0, and the PUTS and DTS items freely loading onto their respective subfactors of urge severity and distress tolerance. In the third step, the bifactor model with urge severity subfactor was examined, with the distress tolerance subfactor fixed at 0. Finally, in the fourth step, the bifactor model with the distress tolerance subfactor was examined, with the urge severity subfactor fixed at 0.

Finally, after establishing a bifactor measurement model of urge intolerance, we examined a structural model where we predicted clinician-rated tic severity and tic impairment by the latent variable urge intolerance among adults with TS.

Models were estimated using weighted least squares mean and variance adjusted (WLSMV) estimation. Model fit was examined using the Comparative Fit Index (CFI), the Standardized Root Mean Square Residual (SRMR), and the Root Mean Square Error of Approximation (RMSEA). Following the precedent established by Hu and Bentler (43), acceptable model fit was defined by CFI values ≥0.95, SRMR values ≤0.08, and RMSEA values ≤0.06. Standardized path coefficients (β) for paths are reported for all models.

## Results

### Characteristics and clinical correlates

Adult participants exhibited a moderate level of tic severity (*M* = 16.22, *SD* = 9.54) and impairment (*M* = 10.00, *SD* = 10.77) (44). Participants reported experiencing premonitory urge severity (*M* = 21.01, *SD* = 7.25) that is comparable with other samples of adults with TS (34). Finally, adults reported moderate levels of distress tolerance (*M* = 37.46, *SD* = 11.41).

There was a moderate relationship between premonitory urge severity and distress tolerance (*r* = 0.39, *p* = 0.001), such that participants who endorsed greater levels of premonitory urges reported lower levels of distress tolerance. Premonitory urges exhibited moderate correlations with clinician-rated tic severity (*r* = 0.37, *p* = 0.002) and tic impairment (*r* = 0.43, *p* < 0.001), such that greater levels of premonitory urges were associated with greater levels of tic severity and impairment. Similarly, distress tolerance was moderately correlated with clinician-rated tic severity (*r* = 0.39, *p* = 0.001) and tic impairment (*r* = 0.39, *p* = 0.001), such that greater levels of tic severity and impairment were associated with lower levels of distress tolerance. Participants' age and sex were not significantly correlated with premonitory urge and distress tolerance ratings. However, participant age exhibited a small association with clinician-rated tic severity (*r* = 0.26, *p* = 0.020) and impairment (*r* = 0.29, *p* = 0.009), such that greater tic severity and impairment was associated with older participant age. Collectively, these findings highlight the modest positive relationships between premonitory urge severity, distress tolerance, tic severity, and tic impairment among adults with TS.

### Evaluating bifactor model of urge intolerance

#### Step 1: General urge intolerance factor, urge severity and distress tolerance subfactors

**Figure 1:**
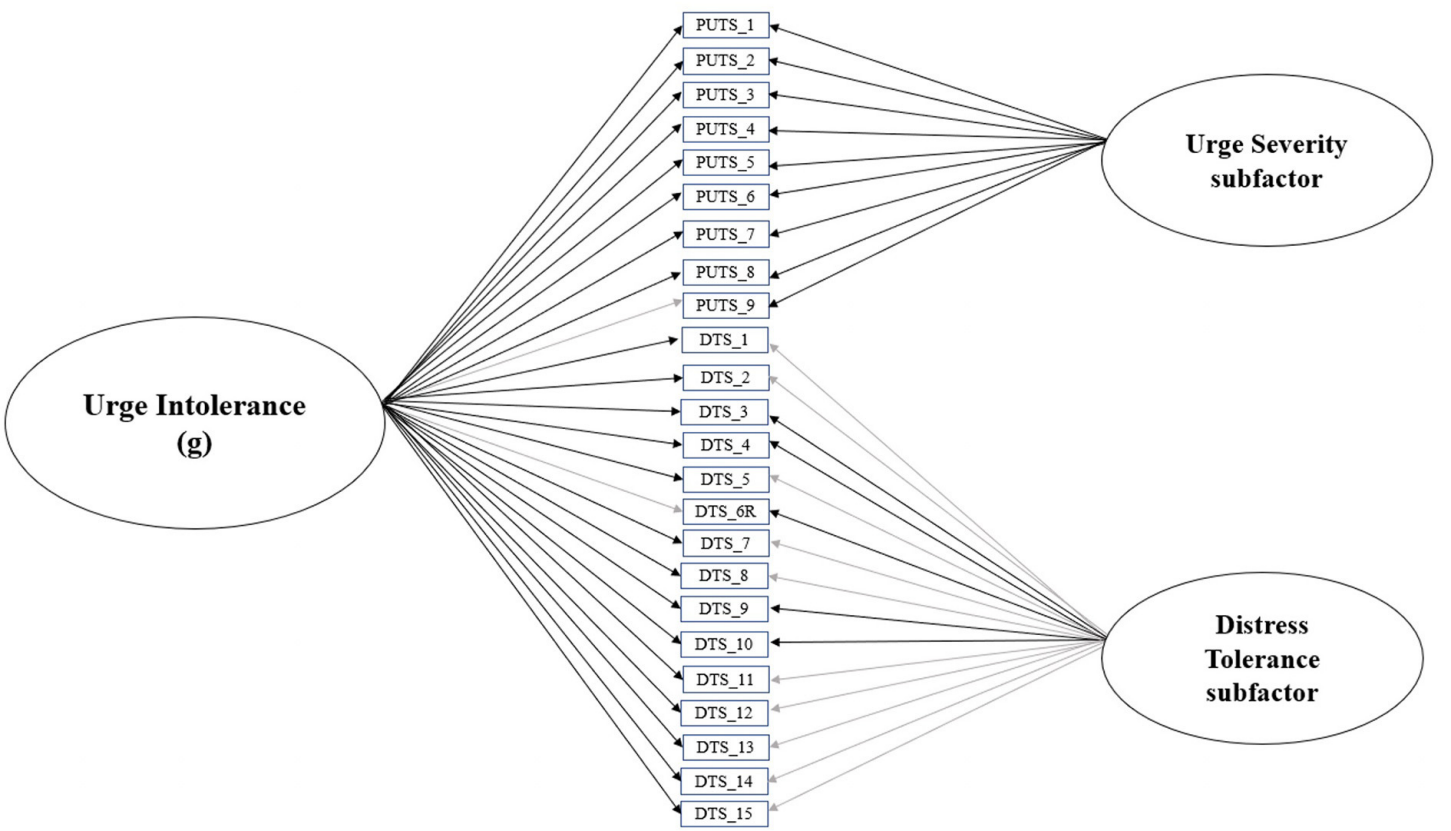
Final bifactor model of urge intolerance, with urge severity and distress tolerance subfactors. Black lines indicate significant standardized item loadings, while gray lines indicate non-significant standardized item loadings.

First, we evaluated the least constrained model (Figure 1)—with all PUTS and DTS items loading onto the general latent factor, urge intolerance, and each items' respective subfactor, urge severity and distress tolerance. Model fit indices were acceptable (CFI = 0.95, RMSEA = 0.08 [90% CI = 0.06–0.09], SRMR = 0.08). Table 1 provides item loadings for the model. As shown in Table 1, the majority of PUTS and DTS scale items loaded onto the general factor of urge intolerance.

**Table 1:** Final retained bifactor model of urge intolerance with premonitory urge and distress tolerance subfactors.

**Item**	**G-factor urge intolerance**	**S-factor premonitory urge**	**S-factor distress tolerance**	**Residual (1-R^2^)**
PUTS_1	0.43 (0.10)*	0.50 (0.09)*		0.56
PUTS_2	0.24 (0.11)*	0.77 (0.07)*		0.35
PUTS_3	0.41 (0.10)*	0.79 (0.06)*		0.22
PUTS_4	0.45 (0.10)*	0.74 (0.06)*		0.24
PUTS_5	0.49 (0.09)*	0.67 (0.07)*		0.31
PUTS_6	0.46 (0.10)*	0.42 (0.09)*		0.61
PUTS_7	0.38 (0.10)*	0.88 (0.05)*		0.09
PUTS_8	0.28 (0.11)*	0.78 (0.06)*		0.32
PUTS_9	0.07 (0.12)	0.48 (0.11)*		0.76
DTS_1	0.77 (0.07)*		0.19 (0.18)	0.37
DTS_2	0.70 (0.09)*		0.32 (0.17)	0.41
DTS_3	0.69 (0.11)*		0.47 (0.17)*	0.30
DTS_4	0.75 (0.10)*		0.39 (0.16)*	0.29
DTS_5	0.70 (0.09)*		0.27 (0.18)	0.44
DTS_6R	0.10 (0.22)		0.86 (0.11)*	0.26
DTS_7	0.45 (0.08)*		0.01 (0.14)	0.80
DTS_8	0.57 (0.08)*		0.01 (0.17)	0.67
DTS_9	0.58 (0.15)*		0.61 (0.14)*	0.29
DTS_10	0.66 (0.12)*		0.45 (0.16)*	0.37
DTS_11	0.71 (0.08)*		0.26 (0.17)	0.43
DTS_12	0.70 (0.08)*		0.15 (0.17)	0.49
DTS_13	0.80 (0.06)*		−0.02 (0.21)	0.36
DTS_14	0.56 (0.11)*		−0.23 (0.16)	0.63
DTS_15	0.83 (0.06)*		0.19 (0.18)	0.28

Standard estimates and (s.e.) for all item loadings in the bifactor model reported.

* Denotes significant loadings in the model (p < 0.05).

#### Step 2: Urge severity and distress tolerance subfactors, general urge intolerance factor fixed at 0

Next, we evaluated whether the exclusion of the general factor of urge intolerance improved the overall model fit. Here, the general factor of urge intolerance was constrained to 0. All items of the PUTS and DTS were exclusively allowed to load onto their respective subfactors of urge severity and distress tolerance. Relative to the full model, model fit statistics deteriorated (CFI = 0.86, RMSEA = 0.12 [90% CI = 0.10–0.13], SRMR = 0.17). Chi-square results indicated that the constrained model (model 2) fit significantly worse than the full model (model 1), χ^2^(24) = 111.94, *p* < 0.001. Stated differently, the full, unconstrained model (with the general urge intolerance factor and the premonitory urge and distress tolerance subfactors) demonstrated significantly better model fit than the partially constrained model with the general urge intolerance factor constrained to 0.

#### Step 3: General urge intolerance factor, urge severity subfactor (distress tolerance fixed at 0)

Next, we evaluated whether the exclusion of the subfactor of distress tolerance improved the overall model fit. Here, the subconstruct of distress tolerance was constrained to 0. Essentially, items on the DTS were only allowed to load onto the general subfactor urge intolerance. Relative to the unconstrained model (model 1), model fit indices deteriorated (CFI = 0.93, RMSEA = 0.09 [90% CI = 0.07–0.10], SRMR = 0.09). Chi-square results indicated that the partially constrained model (model 3) fit significantly worse than the full saturated model (model 1), χ^2^(15) = 54.05, *p* < 0.001. Stated differently, the saturated model (with the general urge intolerance factor and both distress tolerance and premonitory urge subfactors) demonstrated significantly better model fit than the partially constrained model with the distress tolerance subfactor constrained to 0.

#### Step 4: General urge intolerance factor, distress tolerance subfactor (urge severity fixed at 0)

Following this, we evaluated whether the exclusion of the subfactor of urge severity improved the overall model fit. Here, the subconstruct of urge severity was constrained to 0. Essentially, items on the PUTS were only allowed to load onto the general subfactor urge intolerance. Relative to the unconstrained model (model 1), model fit indices deteriorated (CFI = 0.92, RMSEA = 0.09 [90% CI = 0.07–0.11], SRMR = 0.09). Chi-square results indicated that the partially constrained model (model 4) fit significantly worse than the full saturated model (model 1), χ^2^(9) = 25.60, *p* < 0.01. Stated differently, the saturated model (with the general urge intolerance factor and both distress tolerance and premonitory urge subfactors) demonstrated significantly better model fit than the partially constrained model with the urge severity subfactor constrained to 0.

#### Final model

Collectively, these findings suggest that the full bifactor model of urge intolerance (Figure 1), which includes the general urge intolerance factor as well as its premonitory urge and distress tolerance subfactors, is the optimal fit. Consequently, the full bifactor model was used for subsequent analyses.

### Urge intolerance, urge severity, and distress tolerance as predictors of TS severity and impairment

**Figure 2:**
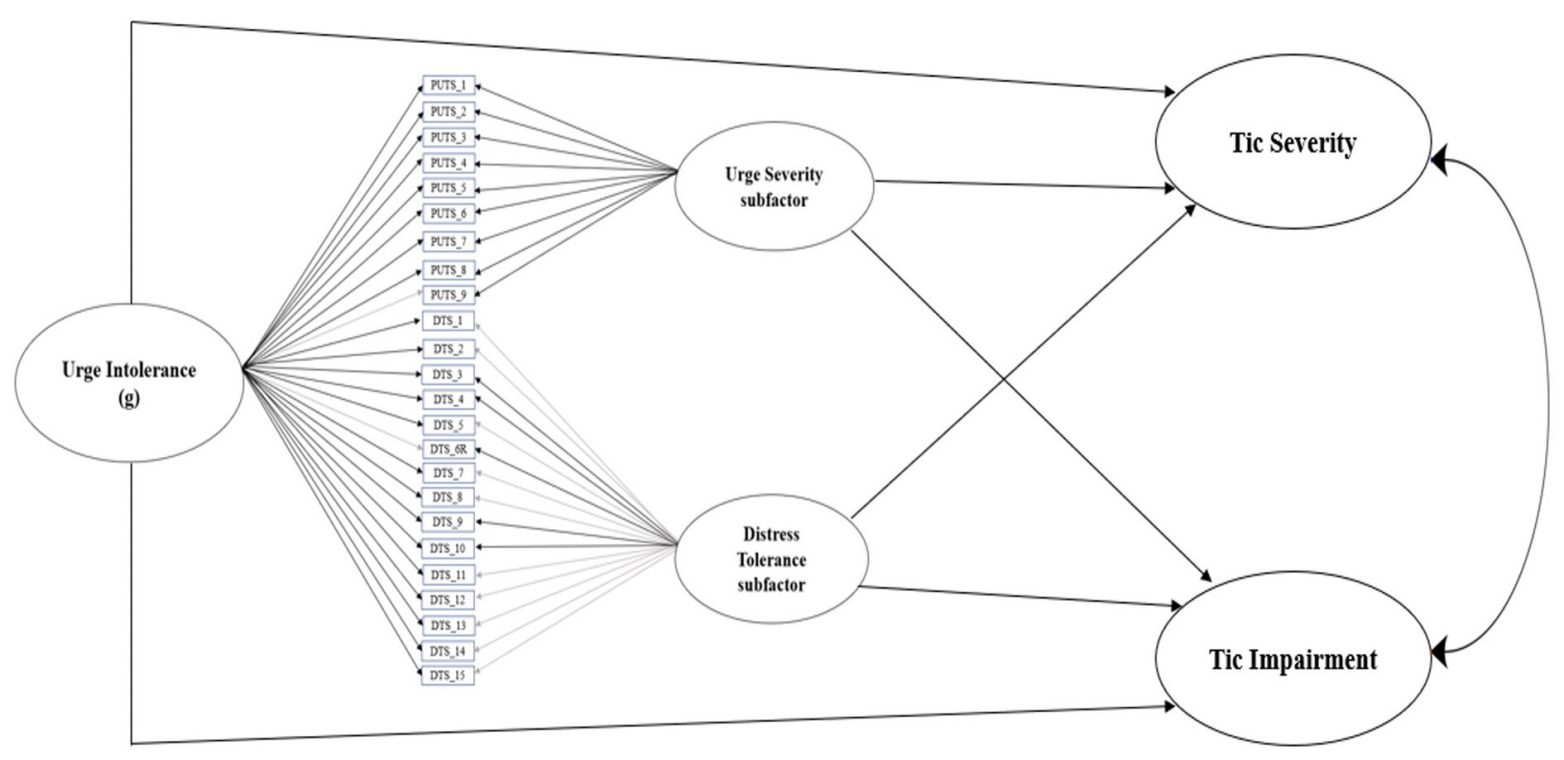
Final bifactor model of urge intolerance, with urge severity and distress tolerance subfactors, predicts YGTSS tic severity and tic impairment. Black lines indicate significant standardized item loadings, while gray lines indicate non-significant standardized item loadings.

Figure 2 illustrates the relationship between the latent construct of urge intolerance, its subfactors premonitory urge severity and distress tolerance, and clinician-rated tic severity and impairment. Table 2 presents standardized path coefficients in the model. Model fit indices were acceptable (CFI = 0.95, RMSEA = 0.07 [90% CI = 0.05–0.08], SRMR = 0.08). Urge intolerance predicted tic severity (β = 0.35, *p* = 0.001) and impairment (β = 0.32, *p* = 0.005). Specifically, greater levels of urge intolerance predicted higher levels of tic severity and impairment.

**Table 2:** Standardized path coefficients for bifactor model of urge intolerance, with premonitory urge and distress tolerance subfactors, predicting YGTSS tic severity and tic impairment.

	**Dependent variable 1: Tic severity**	**Dependent variable 2: Tic impairment**
Urge intolerance	0.35 (0.10)*	0.32 (0.12)*
Premonitory urge	0.23 (0.10)*	0.34 (0.09)*
Distress tolerance	0.21 (0.09)*	0.28 (0.08)*

Standard estimates and (s.e.) for all factor loadings in the bifactor model on dependent variables reported.

* Denotes significant loadings in the model (p < 0.05).

## Discussion

This study examined urge intolerance in adults with TS—a latent construct that encapsulates the ability to tolerate aversive premonitory urges. The bifactor model of the latent construct of urge intolerance was found to be the optimal fit and consisted of a general urge intolerance factor, as well as both premonitory urge and distress tolerance subfactors. In this model, greater levels of urge severity (higher scores on the PUTS) and lower levels of distress tolerance (higher scores on the DTS) contributed to greater levels of urge intolerance (greater difficulty tolerating premonitory urge sensations). Consistent with theorized models, urge intolerance predicted clinician-rated tic severity and tic impairment. Although mixed evidence has been found for the relationship between premonitory urges and tic severity, these findings suggest that the influence of distress tolerance may partly explain the variable relationships premonitory urges and tic severity.

Based on these findings, there are at least two key implications for the field of TS. In regard to the assessment of TS, it is important for clinicians to consider and characterize urge intolerance when conducting evaluations of patients with TS. While this study leveraged existing validated rating scales and used SEM models, there is a need for the development of a standardized rating scale of urge intolerance for individuals with TS. This rating scale could blend items from both the PUTS and DTS, and potentially incorporate other related somatosensory sensations that may be interpreted as urges (e.g., “not just right” sensations). In addition to convergent validity with the PUTS, DTS, and tic severity scales, convergence with objective measures such as tic suppression tasks could also be informative. While empirical testing and validation of such a rating scale would take time, such a standardized scale would allow for a reliable and efficient approach to assess this potentially clinically meaningful construct.

In regard to the behavioral treatment of TS, it is important to consider that urge intolerance was found to predict both tic severity and tic impairment. This suggests that urge intolerance may serve as a novel treatment target to further tic severity reductions among adults with TS. Specifically, CBIT and related behavioral interventions build attention to premonitory urges (i.e., awareness training) and implement behavioral strategies to inhibit tics until premonitory urges are manageable (i.e., competing response training) (16–18). Thus, individuals who have greater difficulty tolerating distressing premonitory urges may have difficulty effectively implementing competing responses in the context of intense premonitory urges. While this possibility requires further empirical investigation, two potential therapeutic strategies exist that could be used to target and improve urge tolerance (i.e., reduce urge intolerance) among individuals with TS to help optimally implement behavioral treatment strategies. One set of skills focuses on mindfulness-based interventions. Gev et al. (45) found that youth with TS experienced reduced levels of tic frequency, distress, and premonitory urges when implementing acceptance-based strategies to address urge phenomena relative to tic suppression strategies. Similarly, Reese and colleagues found that adolescents and adults with TS exhibited improvements in tic severity and functional impairment following a mindfulness-based stress reduction (MBSR) intervention for tics (46, 47). The second set of potential therapeutic strategies focuses on providing distress tolerance skills, which are commonly taught in Dialectical Behavioral Therapy (DBT). This includes training individuals to bring mindful awareness to distressing emotions, physical sensations, and situations and equips them with coping strategies to manage these challenges (48). DBT skills training has been shown to increase distress tolerance capabilities across clinical and non-clinical populations (49, 50). Although future research is essential to determine whether these therapeutic strategies would enhance distress tolerance to premonitory urges (i.e., urge tolerance), such enhancements would have clear implications for reducing tic severity and tic impairment. As urge intolerance is related to TS outcomes for both youth and adults (e.g., tic severity, tic-related impairment) (28), it represents a novel and important therapeutic target. Further research is needed to explore the associations among distress tolerance, urge intolerance, and health-related quality of life among individuals with TS (51). Future work should test treatment strategies that target and improve urge intolerance—particularly during childhood—which may improve patients' clinical trajectories across the lifespan.

Despite the strengths of the present investigation, some limitations exist. First, our bifactor model of the latent construct of urge intolerance was based on subjective, self-report measures (i.e., PUTS, DTS). While these measures are commonly used and facilitate generalizability to other TS studies, they are both self-report ratings. Future research should include a multi-modal assessment of urge intolerance. Alongside self-report ratings, this examination could include clinician-administered measures of premonitory urges (I-PUTS), and standardized tic suppression tasks. This could provide further insights into the relationship between premonitory urges, urge intolerance, and tic severity. It is also important to acknowledge that while many of the instruments utilized in this investigation (i.e., YGTSS, PUTS) have been extensively validated within this clinical population, the DTS has received limited psychometric evaluation in work with adults with TS. Future research is needed to establish the reliability and validity of the DTS within this clinical population. Second, the sample size in the present study was relatively modest for SEM analyses. Despite this, we were able to validate the bifactor model of urge intolerance and identify significant pathways between urge intolerance and TS clinical scales. Finally, the present sample was drawn from a long-term follow-up assessment of a clinical trial for youth with TS. While the sample clinical characteristics are comparable to other samples of adults with TS, future studies should seek to replicate and expand upon findings in both treatment-seeking and non-treatment seeking samples of adults with TS.

In summary, this study provides further evidence for the construct of urge intolerance among patients with TS. Findings highlight the importance of urge intolerance in relation to tic severity and impairment. While behavioral therapies like CBIT remain the front-line treatment for youth and adults with TS (16, 22, 29, 52), patients who do not fully respond to behavioral therapies for tics may benefit from additional therapeutic strategies that target urge intolerance. This could include mindfulness-based interventions and/or distress tolerance skills to enable patients to tolerate distressing premonitory urge sensations. For youngsters with TS, developmentally tailored strategies could be taught alongside CBIT to help youth better tolerate distressing premonitory urges. In turn, youth would be able to optimally implement behavioral strategies (i.e., competing responses) to inhibit tic expression and response to behavioral therapy. This is important because youth who exhibit a treatment response to CBIT in childhood continue to experience therapeutic improvement 11 years later (21) which may be accompanied by other therapeutic benefits as well. Meanwhile for adults with TS, the utilization of strategies targeting urge intolerance could help improve the implementation of behavioral strategies (i.e., competing responses) in the context of treatment. This could lead to greater treatment response rates among those receiving behavior therapy for TS. Ultimately, this line of research holds the potential to provide new insights into the mechanisms underlying tic severity reductions and improve therapeutic outcomes for patients with TS. However, future research is needed to replicate and extend these findings and explore them within the context of treatment.

## Data availability statement

Generated datasets are not available at this time due to forthcoming manuscripts. However, we intend to make the datasets available to qualified investigators upon request once all articles are finalized. Requests to access the datasets should be directed to JM, jfmcguire@jhmi.edu.

## Ethics statement

The studies involving human participants were reviewed and approved by respective institutions, including UCLA Semel Institute for Neuroscience and Human Behavior, Marquette University, and Weill-Cornell Medicine. The patients/participants provided their written informed consent to participate in this study.

## Author contributions

KR contributed to the conceptualization, methodology, formal analysis, validation, and writing–original draft. AD, ER, DW, and JP all contributed to the conceptualization, methodology, formal analysis, and writing–review and editing. JM contributed to the conceptualization, methodology, validation, supervision, and writing–review and editing. All other study authors contributed to the data curation, and/or writing–review and editing. All authors contributed to the article and approved the submitted version.

## Funding

This study was funded by the Tourette Association of America Research Grants Award (JP, Walkup, Woods, & Specht) and the Tourette Association of America Young Investigator Award (KR).

## Conflict of interest

The authors declare that the research was conducted in the absence of any commercial or financial relationships that could be construed as a potential conflict of interest.

## Publisher's note

All claims expressed in this article are solely those of the authors and do not necessarily represent those of their affiliated organizations, or those of the publisher, the editors and the reviewers. Any product that may be evaluated in this article, or claim that may be made by its manufacturer, is not guaranteed or endorsed by the publisher.
